# Urban-Rural and Gender Differences in Cognitive Impairment Among Older Adults in India

**DOI:** 10.1093/geroni/igaf122.2048

**Published:** 2025-12-31

**Authors:** Ratna Patel, Shekhar Chauhan, Waad Ali, Boadi Agyekum

**Affiliations:** International Institute for Population Sciences, Mumbai, Maharashtra, India; Florida State University, Tallahassee, Florida, United States; Sultan Qaboos University, Muscat, Masqat, Oman; University of Ghana, Accra, Central, Ghana

## Abstract

The urban-rural divide in health outcomes has long been a subject of policy discussion, yet cognitive impairment among older adults across urban and rural settings remains underexplored. This study examines urban-rural and gender differences in cognitive impairment among older adults in India, using data from 14,109 individuals aged 60 years and above from the Longitudinal Ageing Study in India (LASI) Wave-1. Logistic regression models with interaction terms were employed to assess how gender (male vs. female) and residence (urban vs. rural) influence cognitive impairment. Additionally, Fairlie decomposition analysis was used to quantify the contribution of key factors to these disparities. Findings reveal that females have a significantly higher likelihood of cognitive impairment than males (OR = 2.63, p < 0.01). Urban residence was associated with a lower likelihood of cognitive impairment compared to rural areas (OR = 0.36, p < 0.01). Interaction analysis showed that females were 1.3 times more likely to report cognitive impairment than males (OR: 1.33; CI: 1.17–1.51), while urban older adults were significantly less likely to report cognitive impairment than their rural counterparts (OR: 0.77; CI: 0.67–0.88). These findings underscore the need for targeted interventions to address cognitive health disparities by gender and residence. Improving access to mental health care and cognitive health resources in rural areas and among older women could help mitigate these inequalities. This study highlights the importance of tailoring cognitive health policies to the socio-cultural and geographic contexts of aging populations in India.

